# Quantitative characterization of thermo-optically modulated microbend loss in silica fibers for high-temperature deep-well telemetry

**DOI:** 10.1038/s41598-026-57752-3

**Published:** 2026-06-13

**Authors:** Haihui Shen, Hu Han, Jianli Liu, Dong Yang

**Affiliations:** 1 The School of Petroleum Engineering, Yangtze University, Wuhan, 430100 Hubei China; 2 The Hubei Key Laboratory of Oil and Gas Drilling and Production Engineering(Yangtze University), Wuhan, 430100 Hubei China; 3 Karamay Vocational & Technical College, Karamay, 834000 Xinjiang China; 4 State Key Laboratory of Low Carbon Catalysis and Carbon Dioxide Utilization, Wuhan, 430100 Hubei China

**Keywords:** High-temperature well, Thermo-optic effect, Microbend loss, Numerical simulation, Spectrum analysis, Engineering, Optics and photonics, Physics

## Abstract

To mitigate the nonlinear amplification of fiber micro-bend loss in high-temperature deep-well environments (303.15 ~ 483.15 K), this study establishes an equivalent refractive index model integrating thermal, mechanical, and optical fields. The model incorporates thermo-optic effects(TOEs) directly into the micro-bend equivalent refractive index expression, facilitating unified modeling of coupled thermal and bending perturbations. Finite element eigenmode analysis was employed to quantify the synergistic effects of bending radius (0.1 ~ 1.0 mm) and temperature on radiation leakage and mode coupling. Results demonstrate that temperature does not act as an independent loss term. Instead, it reconfigures waveguide confinement by modulating the core-cladding refractive index profile, thereby altering mode-coupling pathways. Multimode fiber (MMF) exhibits oscillatory loss governed by inter-modal phase matching, with attenuation coefficients displaying non-monotonic temperature dependence. Conversely, single-mode fiber (SMF) demonstrates threshold-type loss surge at temperatures *T* ≥ 440 K. Spectral and phase analyses reveal that MMF undergoes significant phase decorrelation and topological discontinuity under stochastic mode coupling, whereas SMF maintains phase continuity even under high-loss conditions, demonstrating superior coherence stability. Furthermore, wavelength scanning shows that long wavelengths (> 1.6 μm) significantly enhance SMF micro-bend sensitivity, while the stochastic coupling in MMFs is better suited for short-distance, intensity-modulated telemetry. This study establishes a quantitative correlation between temperature, bending, and wavelength, providing essential physical criteria and engineering guidelines for fiber selection and loss compensation in extreme downhole environments.

## Introduction

With exploration of oil and gas being drawn into deep and non-conventional reservoirs, the downhole sensing systems will have to work under extremely high-temperature and high-pressure (HTHP) conditions. Distributed optical fiber sensing(DFOS) is a newly developed technology that has become fundamental to wellbore monitoring, and its benefits, such as electromagnetic immunity and spatial coverage, have become very critical^1^. Nevertheless, under the conditions of HTHP, the quality of transmission of silica fibers improves dramatically, and the attenuation of the signal seriously worsens the stability of the system over the long run and measurement accuracy^2^.

The attenuation of fibers in complicated downhole conditions is the combined effect of various processes, which include the thermal properties of the material, mechanical stress, and structural inhomogeneities^3^. Amongst them, the most common and the most environmentally sensitive process that is met in field deployments is microbending loss, which has a strong temperature dependence in high-temperature well conditions. The high temperatures have a direct effect of modulating both the refractive index and modulus of elasticity of silica, and also cause or increase the microbending deformations. This establishes close interaction between microbend-induced losses and thermally-induced losses^4,5^, and in this regard, it is necessary to have a thorough mechanistic study of both to enhance the reliability of fiber-optic telemetry under extreme conditions in deep wells.

The study of the microbend loss has had a progressive transition into statistical theories of loss at the classical level and high-fidelity numerical techniques. The mode-coupling equations of Marcuse^6^ defined the basic image: when microbends are introduced, loss is created when the guided modes are converted into radiation modes. Based on this, Gloge used the power spectral density (PSD) theory to consider microbending as a stationary random process, showing that the magnitude of attenuation is determined by the spectral magnitude of the axial deformation at particular spatial frequencies. Here, we have within this framework the Gaussian power spectra of general random microbends^7^ and exponential models, which are a better fit of the deformations caused by rough contact surfaces in real-world deployments^8^. In more recent developments, the finite-difference beam propagation method (FD-BPM)^9^ and finite element method (FEM)^10^ have been able to achieve the rigorous simulation of complex deformation profiles and allow optimization of fiber design parameters in a wide operating conditions regime compared to analytical methods.

Simultaneously, FEM and beam propagation have emerged as leading tools in the study of loss due to temperature in optical fibers^11^. Common modeling procedures are the development of the thermomechanical models to compute the distribution of residual stress, complemented by electromagnetic modeling to plot the mode field evolution. In the case of MMFs, high temperatures are discovered to increase mode coupling and to hasten the leakage of higher modes into the cladding^12^. In the case of SMFs, studies have been done on the thermal sensitivity of the basic mode LP01, with increasing temperature increasing the mode field diameter (MFD) and decreasing the mode confinement, becoming more vulnerable to perturbations of microbending^13^.

In spite of these developments, there are still critical gaps. At well depths greater than 6000 m, the temperature is more than 473 K and the pressure is more than 150 MPa^14^, but even the systematic experimental measurements of the principal thermophysical properties of silica, especially of the TOE values^15,16^, under extreme conditions are limited. The vast majority of existing simulation studies are thus based on parameters that are extrapolated from the ambient conditions, and thus create large deviations between the theoretical outcome and the true downhole results. This dependence badly limits the use of existing models in both the analysis of mechanistic and the prediction of engineering.

In coupled HTHP regimes, local curvature perturbations are further enhanced by the degradation of mechanical properties and thermal expansion mismatches to produce nonlinearly coupled mode-coupling processes that are beyond the capability of weak-perturbation theories to describe^17^. Multi-scale and multi-frequency microbending perturbations are caused by the net effect of non-uniform external loads and internal thermal stresses, and the strong interaction between thermal and geometric effects makes quantitative decoupling a focus of the physical modelling. The technical challenge of reproducing ultra-deep-well conditions deeper than 10 km in the laboratory is challenging, and the current experimental systems fail to provide the various conditions needed to support stable high pressure and simultaneously provide controllable microbending loads and high-resolution real-time measurements of losses^18^.

Above all, the only area that is lacking is the fact that although there is prior research on microbending and TOE as independent variables, a physical system that quantitatively measures both variables under severe conditions in a downhole setting does not exist. The consideration of thermal and bending losses as linearly additive does not have the capability to model non-monotonic attenuation curves, threshold transitions, and progressive signal losses, being a constant phenomenon in downhole field work, which severely limits the predictive capability of current models.

While macro-bending research^19^ has elucidated the loss mechanisms associated with large-scale geometric deformation, this study focuses on stochastic, micro-scale perturbations driven by complex mechanical stresses in deep-well environments. It emphasizes the quantitative analysis of the TOE and modal phase fading. As a foundational step, this study primarily focuses on steady-state analysis. In order to overcome these difficulties, we would recommend a multiphysics theory where we incorporate TOE directly on the microbend equivalent refractive index formulation in a coupled thermo-mechanical-optical numerical model. We show that the attenuation is not additively attenuated by thermal effects but is basically restructured by them, by reorganizing the distribution of refractive indices of the core and cladding and changing the dynamics of radiation leakage that accompanies microbending. We discover quantitative attenuation behaviour of SMFs and MMFs to coupled thermal and microbending perturbations, and in both the PSD and phase spectrum analysis, we find inherent differences in signal stability and coherence between the two fiber types. MMFs have oscillatory loss characteristics that are characterized by modal interference and phase-topology disruption, and SMFs have threshold-like attenuation that maintains phase integrity. The findings aid a sound physical foundation of fiber choice, wavelength design, and signal restoring plans in the deep-well distributed sensing systems.

### Principles of temperature-dependent bending loss in optical fibers

#### Principles of fiber optic bending

In high-pressure downhole environments, micro-bending disturbances sustained by fiber-optic sensing systems constitute a complex multiphysics coupling problem. The crux of this problem lies in the transmission of external hydrostatic and non-uniform confining pressures to the silica fiber core via structural mechanical pathways. Typically, specialized polymer coatings such as acrylates, polyimides, or fluoropolymers possess a low elastic modulus ranging from several megapascals to several gigapascals and a high Poisson’s ratio between 0.4 and 0.5, which indicates near-incompressibility. In contrast, the silica core exhibits a much higher elastic modulus of approximately 72 GPa and a lower Poisson’s ratio of 0.17^20^. At the coating–silica interface, material anisotropy or geometric asymmetries—such as fiber ellipticity or non-uniform coating thickness—converts uniform radial pressure into non-uniform transverse loads, ultimately inducing micro-bending deformations with distinct spatial spectral characteristics^20^.

**Fig. 1:**
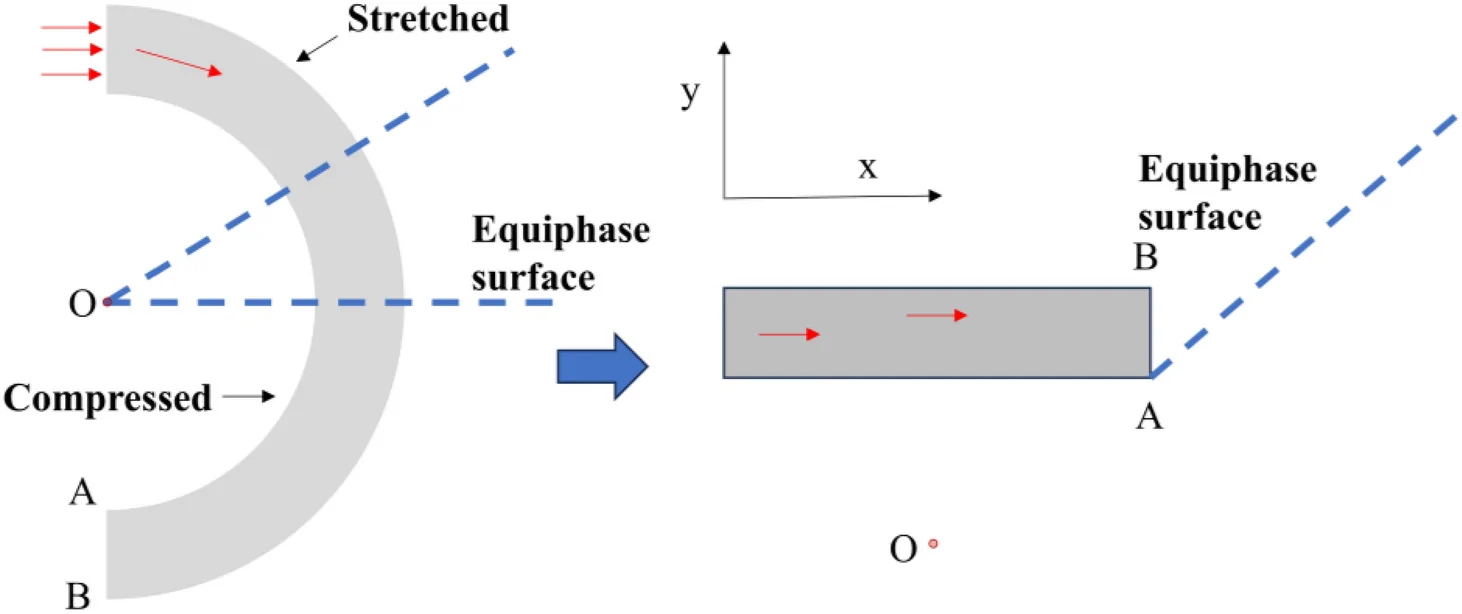
The schematic diagram of conformal mapping for a bent optical fiber.

To describe the propagation in a bent fiber, we employ the equivalent refractive index method (Fig. 1). For a fiber with a bending radius *R*, we define a local Cartesian coordinate system where the x-axis is the unbent fiber axis and the *y*-axis points radially outward from the curvature center. Consequently, bending causes the geometric path length to increase in the outer region (*y* > 0) and decrease in the inner region (*y* < 0), thereby modulating the phase accumulation across the fiber cross-section.

For fibers with a large bending radius *R* relative to their transverse dimensions, conformal mapping allows for the representation of a curved waveguide as an equivalent straight one by introducing a coordinate-dependent refractive index correction^22^. Grounded in the principle of phase conservation, this approach ensures the consistency of propagation constants across the transformation. Consequently, the equivalent refractive index at a given radial position can be expressed through the following first-order approximation:

1 $$n(x,y) = n_{0} \exp (\frac{y}{R}) \approx n_{0} (1 + \frac{y}{R})$$

In this model, *n*_0_ is the first radial refractive index of the straight fiber. The term describes the curvature-induced correction to the local propagation constant, in which the ensuing transverse index gradient, which is a consequence of the change in index across the cross-section of the fiber, changes the mode field distribution and adjusts mode coupling. This is a mechanism that forms the geometric basis of bending loss. Two major simplifications that must be satisfied to make the model applicable are that the curvature should be planar and gradually varying, and other higher-order curvature effects and modulation of the index by photoelastic (stress-induced) effects should be ignored.

The second form of fiber bending is due to the effect of stress. With the bending of the fiber, the outer is stretched, and the inner is compressed, hence the refractive index is changed because of the stress-optical effect^23^. On the outer surface (Side B), positive strain (*ξ* > 0) is induced, which normally decreases the refractive index. On the inner side (Side A), on the other hand, compression causes negative strain (*ξ* < 0), which usually enhances the refractive index. The refractive index of the fiber, therefore, varies in the following manner:

2 $$\Delta n = n_{i} - n_{0} = - C_{2} \sigma_{i} - C_{1} (\sigma_{j} + \sigma_{k} )$$

where *σ*_i,j,k_ represent the stress tensor elements. Under bending, the primary axial strain is defined as *ξ*_*z*_ = *y*/*R*, with *y* being the radial distance from the bending axis. Assuming an isotropic material, the stress components follow Hooke’s law: *s*_z_ = *C*_11_*ξ*_*z*_ and *s*_*x*_ = *s*_*y*_ = *C*_12_*ξ*_*z*_. Here, *C*_11_ and *C*_12_ are the elastic stiffness constants, taking values of approximately 86.3GPa and 10.3GPa for silica, respectively^24^. The resulting stress-induced refractive index variation, *n*_s_(*x*,*y*), is then expressed as:

3 $$n_{s} (x,y) = n_{0} + \vartriangle n(x,y) = n_{0} - \frac{y}{R}[C_{2} C_{12} + C_{1} (C_{12} + C_{11} )]$$

The stress-induced anisotropic refractive index, after neglecting higher-order terms, is thus defined as:

4 $$n(x,y) = n_{s} (x,y) \times (1 + \frac{y}{R}) \approx n_{0} (1 + \frac{y}{R}[1 - \frac{1}{{n_{0} }}(C_{2} C_{12} + C_{1} (C_{12} + C_{11} ))])$$

Rewriting the above expression to align with Eq. (1), and letting:

5 $$\frac{1}{{R_{{{\mathrm{eff}}}} }} = \frac{1}{R}[1 - \frac{1}{{n_{0} }}(C_{2} C_{12} + C_{1} (C_{12} + C_{11} ))])$$

After simplification, the above expression yields:

6 $$R_{{{\mathrm{eff}}}} \approx \frac{{n_{0} R}}{{n_{0} - 0.414}}$$

Under the conditions of a homogeneous fused silica medium, and ignoring the effect of coating (because of the ambiguity of the parameters), the resultant effective bending radius *R*_eff_ is applicable uniformly to the fiber cross-section. This correction is determined by the intrinsic elasto-optic constants of silica, which do not depend on the mode, hence *R*_eff_ is the same with both SMF and MMF. Approximating the refractive index of silica as *n*_0_≈1.45^25^, Eq. (6) yields *R*_eff_≈1.40R. The finding proves that the stress-optical effect enhances the effective curvature radius, which can be used to suppress bending loss.

Taking into account the geometric and stress-optic terms that are caused by the bending of the fibers, the overall expression of the final change in refractive index is obtained as follows:

7 $$n(x,y) = n_{0} (1 + \frac{y}{1.4R})$$

where the term *y*/(1.40*R*) represents the effective bending correction. Consequently, the complicated 3D curved structure is essentially simplified to the study of the simple straight waveguide with an altered refractive index structure. Such a change makes it mathematically and numerically manageable and allows effective theoretical and simulation work.

Although this study employs a 2D cross-sectional approximation, it relies on the adiabatic assumption that longitudinal curvature variations remain negligible. This approach facilitates a focused analysis of the multidomain coupling between thermal perturbations and modal leakage, which remains the primary cause of total loss under stable micro-bending conditions.

### Bending principles of optical fibers under high-temperature conditions

The thermally induced attenuation and mechanical bending loss are the two main sources of bending loss in optical fibers in downhole high-temperature applications. Most importantly, the effectiveness of these two mechanisms is mutually dependent, which makes them the most significant factor contributing to the loss.

#### Principles of high-temperature attenuation in optical fibers

The temperatures down the hole are usually linear with depth and controlled by a geothermal gradient of about 3 K/100 m in the absence of any localized thermal effects. In very deep wells, the working temperature is often higher than 423.25 K or even 473.15 K^26^. In such high-temperature conditions, thermal expansion and polarizability effects are the most important in variations in the refractive index of the fiber. The obtained thermo-optic correlation can be expressed as:

8 $$\frac{dn}{{dT}} = - \gamma (\frac{\rho \partial n}{{\partial \rho }})_{T} + (\frac{\partial n}{{\partial T}})_{{\rho^{,} }}$$

where *ρ* is the density and *γ* is the thermal expansion coefficient. The formula separates the variation of the index due to the change of density (thermal expansion) and the change in polarizability of the same density^27^. The effect of thermal expansion (10^−7^) is not significant in silica fibers in comparison to the effect of polarizability (10^−5^). Hence, polarizability is the main factor that dictates the development of the refractive index.

Adopting a polarizability thermal coefficient of *δ* = 2.0 × 10^−5^ K^−1^, the thermo-optic coefficient(TOC) d*n*/d*T* is calculated via Eq. (9). The final temperature-dependent refractive index profile is then given by Eq. (10), assuming a reference temperature *T*_0_ = 293.15 K:

9 $$\frac{dn}{{dT}} = \frac{{(n^{2} - 1)(n^{2} + 2)}}{6n} \cdot \delta$$

10 $$n(T) = n_{0} + \frac{dn}{{dT}}(T - T_{0} )$$

#### The combined effects of bending and elevated temperature on the refractive index of optical fibers

Bending perturbs the waveguide’s axisymmetry, causing energy from the fundamental guided mode to couple into radiative modes, thereby inducing loss. This attenuation is highly nonlinear with respect to the bending radius *R*. Conversely, thermally induced loss is governed by the TOE. Temperature variations alter the refractive index profile of the fiber, which in turn modulates fundamental waveguide parameters such as the normalized frequency $V = k_{0} a\sqrt {n_{{{\mathrm{core}}}}^{2} - n_{{{\mathrm{clad}}}}^{2} }$^28^.

According to Marcuse’s theory, the bending loss coefficient in an optical fiber with a bend radius *R* can be expressed as follows:

11 $$2\alpha_{C} = \frac{{\sqrt \pi U^{2} }}{{e_{i} W^{{{3 \mathord{\left/ {\vphantom {3 2}} \right. \kern-0pt} 2}}} \sqrt {aR} V^{2} K_{i - 1} (W)K_{i + 1} (W)}}\exp ( - \frac{{2W^{3} }}{{3a^{2} \beta^{2} }}\frac{R}{a})$$

where $U = a\sqrt {k^{2} n_{{{\mathrm{core}}}}^{2} - \beta }$ and $W = a\sqrt {\beta^{2} - k^{2} n_{{{\mathrm{clad}}}}^{2} }$ are the normalized transverse phase and attenuation constants, respectively. The parameters $k = {{2\pi } \mathord{\left/ {\vphantom {{2\pi } \lambda }} \right. \kern-0pt} \lambda }$, *a* and *β*, denote the free-space wavenumber, core radius, and axial propagation constant. *K*_i_ represents the modified Bessel function of the second kind of order *i*, with the Neumann factor defined as *e*_i_ = 2 for *i* = 0 and *e*_i_ = 1 for *i* ≠ 0. As a result, temperature variations have an inherent impact on *U*, *W*, and *V* because they change the properties of the constituent material.

And in contrast to classical additive noises (e.g., thermal or shot noise), where the sources of the noise are independent and superposable, there is no separation between sources in this case, and temperature and bending signals are coupled. Instead of directly contributing to the loss, the temperature does play the role of modulating the microbending response function^29^. In this case, the bending is the carrier (characterizing the basic attenuation profile—usually exponential decay), and the temperature is the modulator, adjusting the level of the loss by changing the refractive index. Under this modulation mechanism, the variation in material refractive index due to thermal and bending perturbations combined is determined as:

12 $$n_{{sio_{2} }} (x,y,T) = n_{0} (1 + \frac{dn}{{dT}}(T - T_{0} ) + \frac{y}{{R_{eff} }})$$

Though Eq. (11) presumes weak guidance, it still holds for the MMF analyzed here, since the relative index difference Δ(~ 1.5%]) falls within the standard bounds of the approximation. Beyond that, this analytical form faithfully captures the regular modulation of the loss function by temperature-induced parameter shifts—a behavior corroborated by our full-wave simulations.

It is worth noting that the present model adopts a planar adiabatic assumption to isolate the core thermo-optic modulation mechanisms that actual downhole deployments entail multi-scale, stochastic 3D twisting. Such non-planar trajectories break spatial symmetry, inducing a geometric topological phase (i.e., the Berry phase) and twist-induced elasto-optic birefringence. These phenomena lift the degeneracy of orthogonal core modes in SMFs, triggering localized polarization mode coupling. However, under standard downhole structural constraints where the twist rate is orders of magnitude smaller than the local curvature, these 3D topological effects manifest as higher-order perturbations. Over extended telemetry distances, these effects undergo statistical averaging, enabling the planar model to retain high fidelity in predicting macroscopic loss trends^30^. Incorporating full 3D stochastic twisting tensors into the phase continuity matrix represents a promising avenue for future multi-scale extensions of this framework.

### The development of models

#### The model parameters and grid

The simulation parameters are summarized in Table 1. A sharp profile of refractive index is achieved through the use of a step-index fiber model, and thus, rigorous modal analysis is enabled. The SMF is made of Ge-doped silica core (n = 1.44 ~ 1.47) and pure silica cladding (n = 1.44 ~ 1.46). The MMF has the same cladding material but with a larger core refractive index (n = 1.47 ~ 1.49)^31^. In order to simulate normal downhole conditions, operating temperatures are between 303.15 K and 483.15 K. In addition, the bending radius is adjusted to a range of 0.1 mm to 1.0 mm to determine theoretical failure limits in excessive downhole conditions. The TOCs d*n*/d*T* are determined in correspondence with the following equations (see Eq. (9)) as follows: 1.0 × 10^−5^ K^−1^ as the cladding of the two fibers, 1.2 × 10⁻^5^ K^−1^ as the core of the SMF, and 1.5 × 10^−5^ K^−1^ as the core of the MMF.

**Table 1:** The parameters of models.

Physical quantity	Value	Description
λ	1.2um-2.0um	Wavelength
R	0.1 ~ 1.0mm	Bending radius
*T*_1_	303.15K ~ 483.15K	Operating Temperature
*n*^s^_clad_/*n*^m^_clad_	1.4565	Initial refractive index of MMF/SMF cladding
*n*^s^_core_	1.461	Initial refractive index of the SMF core
*n*^m^_core_	1.48	Initial refractive index of MMF core
*d*_1_	1.2 × 10^-5^K^-1^	TOC of SMF core
*d*_2_	1.0 × 10^-5^K^-1^	TOC of SMF/MMF cladding
*d*_3_	1.5 × 10^-5^K^-1^	TOC of MMF core
*R*_core1_	25um	MMF core radius
*R*_core2_	4.15um	SMF core radius
*R*_clad_	62.5um	Fiber optic cladding radius

The studies of the TOE are mainly based on experimental characterization. The Tsukuba Electronics and Communications Laboratory explored GeO₂-doped silica glass in 613-808 K, with a TOE of about 1.3 × 10⁻^5^ K^-1^ to 1.4 × 10^−5^ K^-1^ at 0.5894 μm^32^. The group at the University of Electronic Science and Technology of China measured a TOC of 1.178 × 10^−5^ K^-1^with step-index fibers having GeO_2_-doped core and pure silica cladding ^33^, and researchers at the Beijing Institute of Technology measured coefficients between 1.09 × 10⁻^5^ K^-1^ and 1.417 × 10^−5^ K^-1^ between 0–1273 K^34^. These results, despite the fact that the material composition, wavelength, and temperature regime differ, show considerable consistency and represent a strong reference point to use in correcting refractive indices at the high-temperature regime. The paper, as a result, incorporates TOE as a temperature-dependent parameter into the equivalent model of the refractive index, with the microbending term, to develop a comprehensive perturbation framework, which is consistent with experimental observations.

Although the TOC of silica glass exhibits slight temperature dependence over wide ranges, linear approximations (*d*_1_, *d*_2_, *d*_3_) are employed because variations remain within a minimal margin (under 10%) up to 483 K. This approach is consistent with established empirical data^32–34^. By adopting these approximations, the model ensures a balance between computational efficiency and physical accuracy in characterizing the steady-state thermal response.

**Fig. 2:**
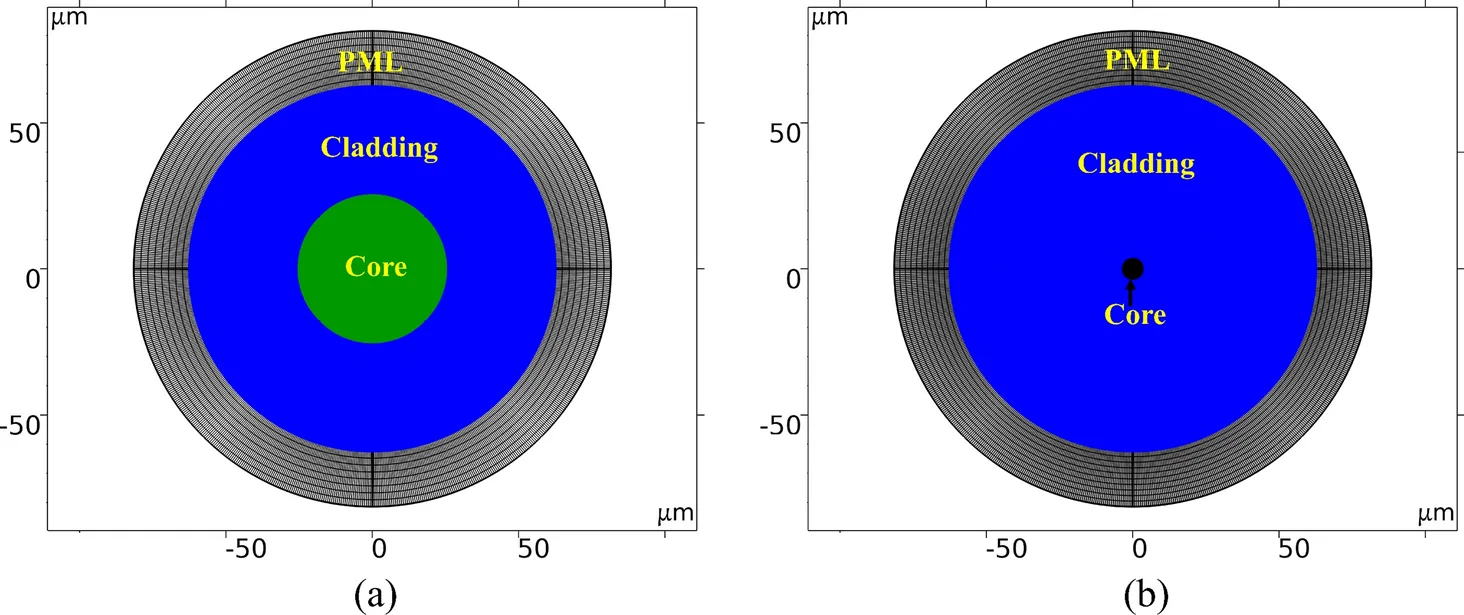
The 2D geometric models of optical fibers, (**a**) MMF; (**b**) SMF.

This study utilizes the COMSOL Multiphysics platform (Wave Optics Module) to establish a 2D frequency-domain electromagnetic wave model of the optical fiber cross-section to quantitatively analyze the thermal response characteristics under pre-existing micro-bending conditions. Based on the geometric parameters from Table 1, an eigenmode analysis is conducted on the cross-sectional structure illustrated in Fig. 2. By solving for the complex effective refractive index (*n*_eff_ = *n*’ + i*n*’’), the radiation loss coefficient (*α* = 2*k*_0_*n*’’, where *k*_0_ is the free-space wavenumber) is derived. Temperature and equivalent curvature perturbations are introduced via a parametric sweep to map the loss–temperature response, while the equivalent refractive index is updated according to Eq. (12).

Previous literature indicates that during the evaluation of fiber bending or thermal losses, the core-guided mode and the cladding whispering gallery mode (WGM) undergo coupled interference. This interference—either constructive or destructive—depends heavily on the operating wavelength, bending radius, and cladding refractive index. Fluctuations in temperature modulate these parameters through thermo-optic and thermal expansion effects, thereby significantly shifting the coupling state between the core and WGM modes. Such oscillatory and nonlinear characteristics compromise the stability and reliability of fiber-optic transmission and sensing systems. Specifically, they complicate the calibration of the loss–temperature curve and induce sensitivity fluctuations within narrow thermal intervals.

Furthermore, because this study focuses on intrinsic optical coupling rather than mechanical load transfer, the geometric model deliberately omits the outer polymer coating, relying instead on equivalent stress-optic and curvature terms to capture thermomechanical stresses induced by coefficient of thermal expansion (CTE) mismatches (e.g., between silica and polyimide). From a numerical modeling perspective, this simplification represents a critical trade-off between physical fidelity and computational convergence. Due to the high imaginary refractive index of the polymer, directly meshing the highly absorptive coating within a frequency-domain eigenmode solver introduces severe non-physical spurious modes and triggers convergence failure. Decoupling the mechanical load as a parametric boundary perturbation and positioning the perfectly matched layer (PML) directly at the cladding interface eliminates these numerical artifacts^35^, optimizing computational convergence while capturing the intrinsic core-cladding WGM resonance behavior with high fidelity. Calculations are performed on a 2D cross-section, applying an equivalent uniform approximation in the axial direction (with a default thickness of 1 m). This approximation is suitable for scenarios characterized by gradually varying axial curvature. This modeling framework enables reproducible, convergent calculations of coupled thermal–micro-bending losses while ensuring physical interpretability.

The arrangement of the computational mesh is depicted in Fig. 2. Given that the optical mode is mainly enclosed within the core and moves outwards on bending, refined meshing is used in the core and outer cladding areas, with the size of the elements limited to *λ*/*G*, where *G* is the mesh refinement factor. The finer mesh (having the largest element size *λ*/3 ) is used in the inner cladding where the field intensity is negligible. A 15-layered mapped mesh of the PML domain is used to provide good absorption of outgoing radiative waves.

### The model convergence conditions

**Fig. 3:**
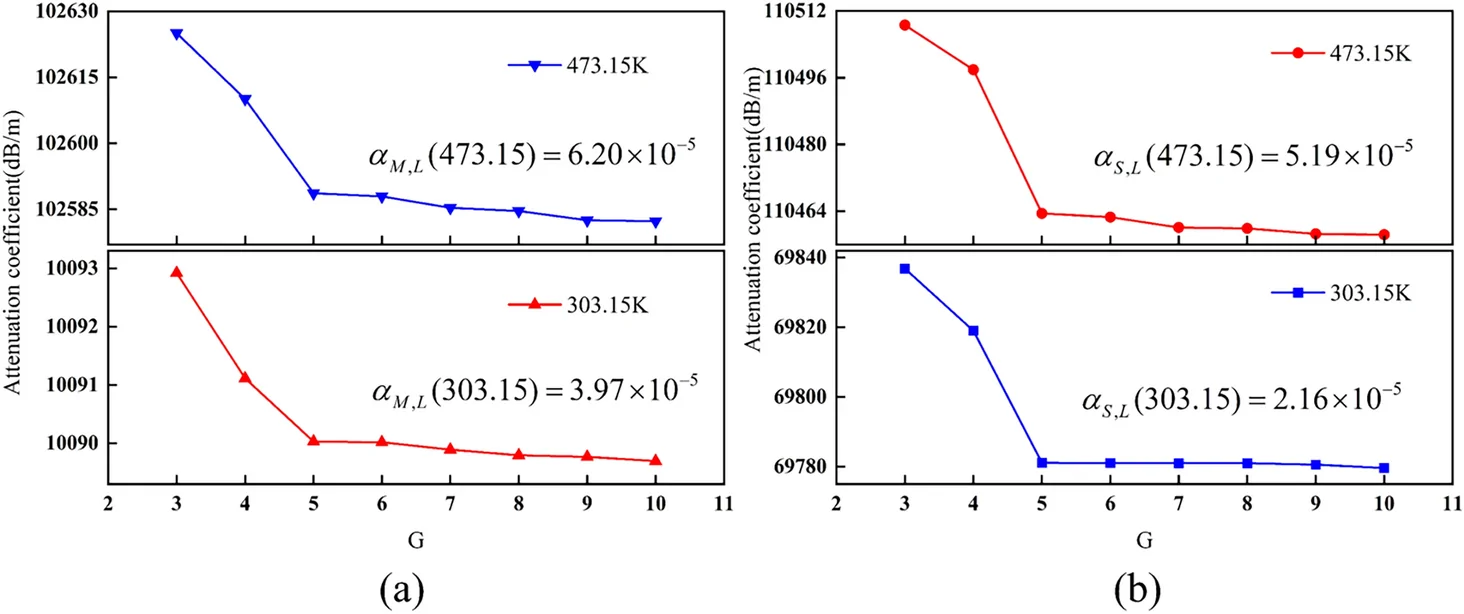
The convergence of *G*, (**a**) the relationship between MMF model grid parameters and attenuation coefficient; (**b**) the relationship between SMF model grid parameters and attenuation coefficient.

**Fig. 4:**
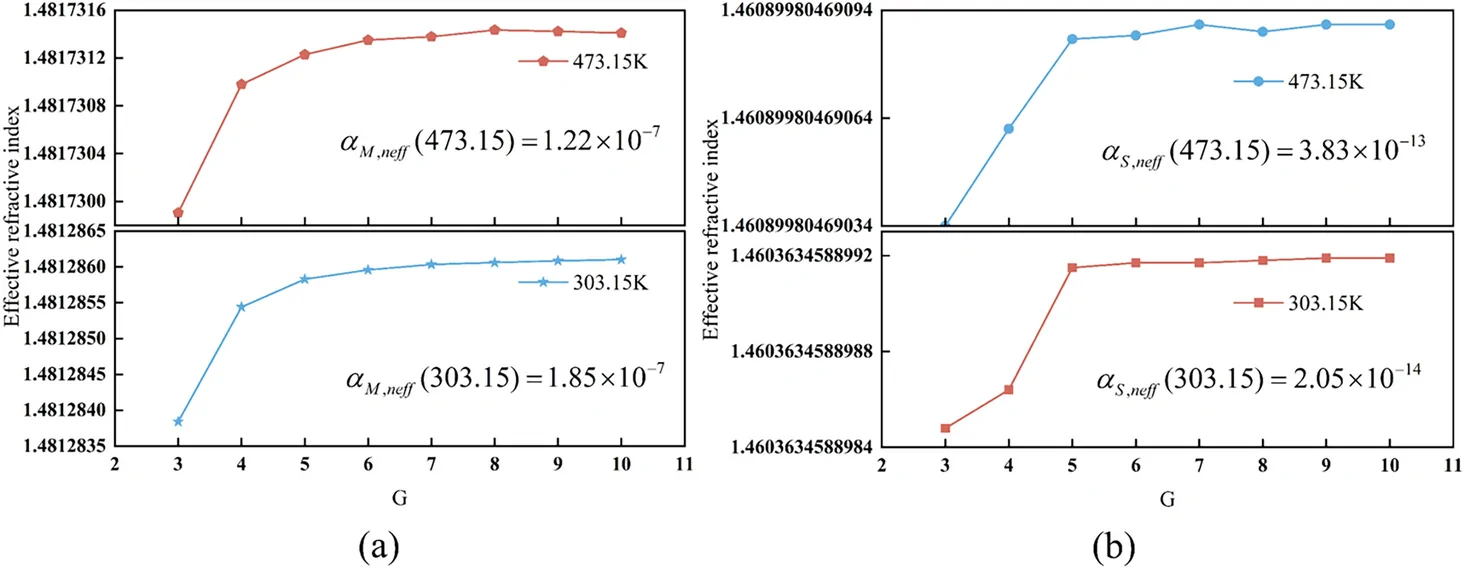
The relationship between G and effective refractive index, (**a**) the relationship between MMF model grid parameters and effective refractive index, (**b**) the relationship between SMF model grid parameters and effective refractive index.

Figures 3 and 4 are model convergence plots versus mesh parameter *G*. Both attenuation coefficient and effective refractive index stabilize at *G* ≥ 5. Figure 3 indicates that the relative deviation of the attenuation coefficient when benchmarked to *G* = 10 decreases to the order of 10^−5^ over all test temperatures.

The effective index variation of Fig. 4 is still less than 10^−7^ of MMF and 10^−13^ of SMF versus *G* = 10. These findings verify that no significant accuracy gain would be achieved by an additional mesh refinement so as to meet the mesh independence criterion. Based on this, the *G* = 5 is taken as the basis of all further computations.

**Fig. 5:**
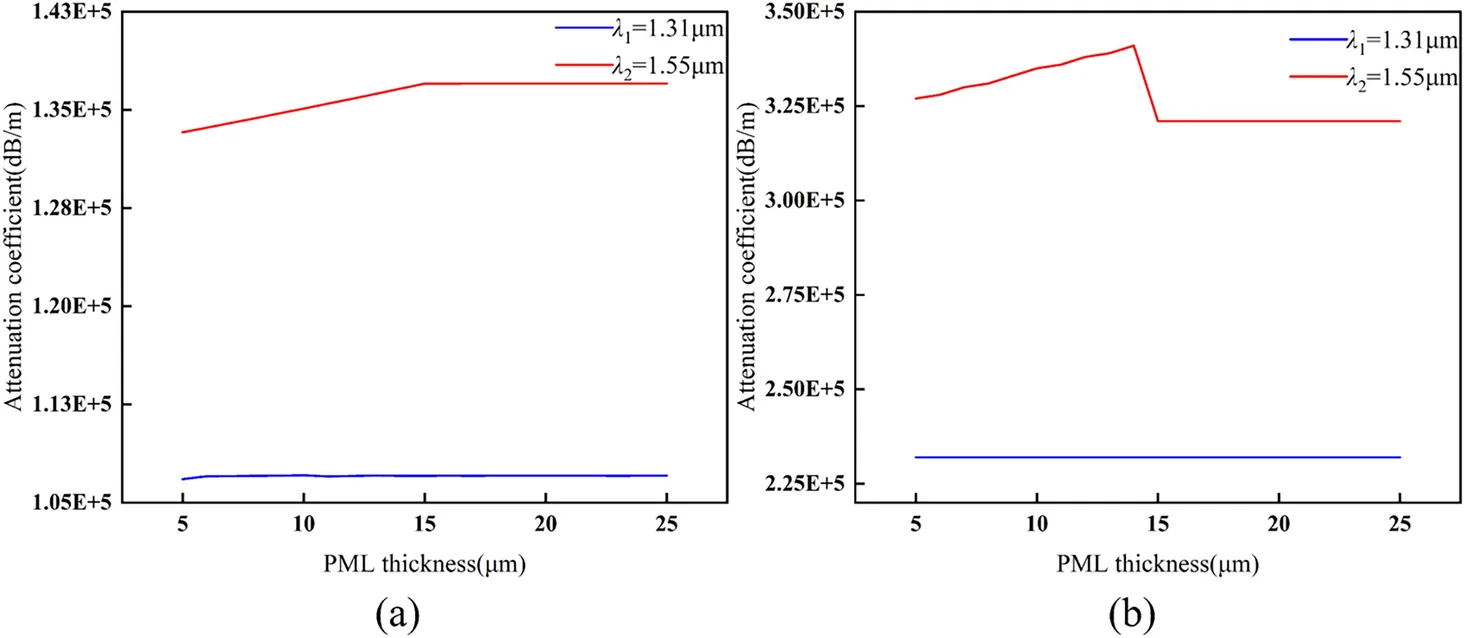
The effect of PML thickness on fiber model attenuation coefficient at two operating wavelengths, (**a**) the attenuation coefficient of MMF with varying PML thicknesses, (**b**) the attenuation coefficient of SMF with varying PML thicknesses.

To suppress non-physical reflections, the model utilizes a perfectly matched layer (PML) integrated with an outer scattering boundary. The real part of the PML refractive index matches that of the cladding, whereas its imaginary part and thickness dictate the modal power attenuation within this region. Figure 5 illustrates the impact of PML thickness on convergence across two operating wavelengths. For both fiber types, at a PML thickness of 15 μm, the relative change in the MMF loss coefficient (referenced to a 24 μm thickness) is 3% at 1.31 μm and 2% at 1.55 μm. For the SMF, these relative changes are 2% and 1%, respectively. In finite element analysis (FEA), a relative change of less than 5% in key responses due to PML variations indicates sufficient thickness for numerical convergence^36^. Consequently, the models have a PML thickness of 15 μm in order to achieve convergence between the modal imaginary effective refractive index.

### The model validity analysis

**Fig. 6:**
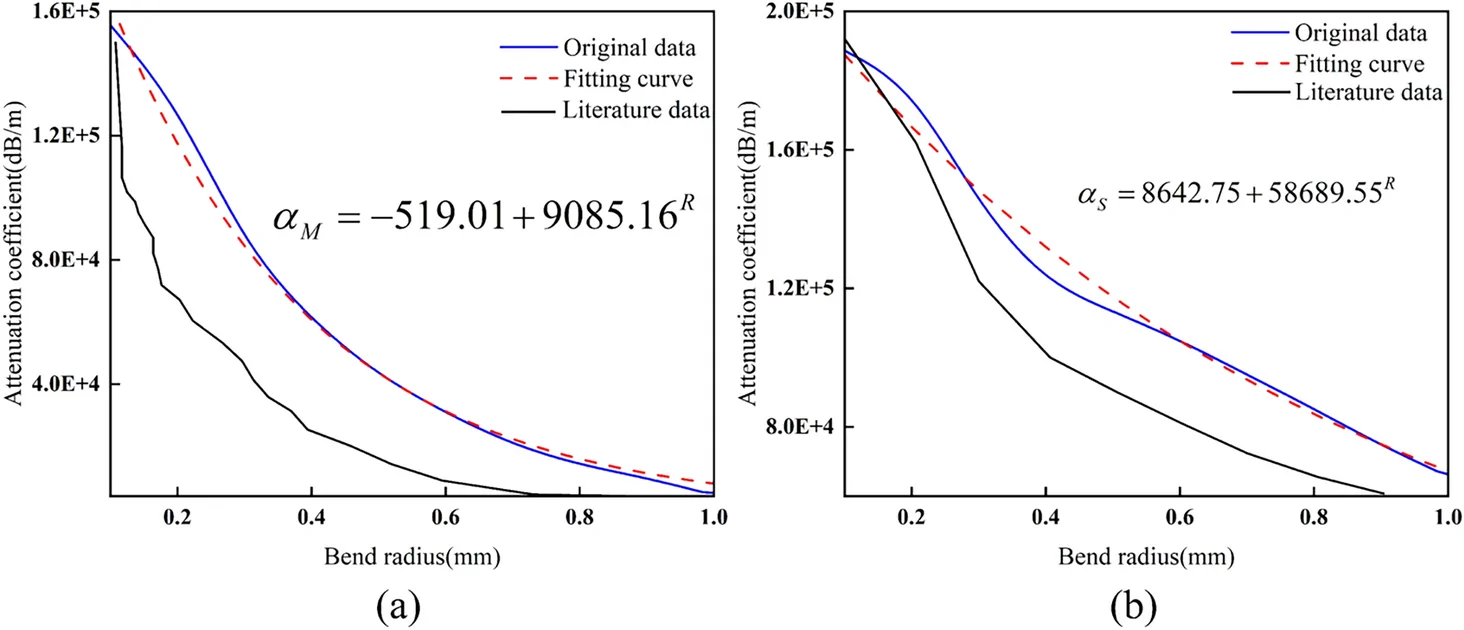
The trend of fiber attenuation coefficient with bending radii, (**a**) the trend of MMF attenuation coefficient with bending radii, (**b**) the trend of SMF attenuation coefficient with bending radii.

Figure 6 shows a dependence of MMF and SMF attenuation coefficients on bending radius *R* at 1.55 μm based on the equivalent refractive index model of the form of Eq. (7). When *R* is smaller, less confinement of transverse modes will lead to an exponentially increasing attenuation coefficient as a result of increased leakage of the radiation. The reproduction of simulated behavior is accurate across the range tested, as confirmed by an empirical exponential fit (*R*^2^ = 0.99). The results of simulations indicate that the published experimental data^9,37^ agree with strong quantitative accuracy in magnitude and trend and relative error (|*α*_sim_-*α*_exp_|/*α*_exp_) of 3.5% and 1.8% of MMF and SMF at minimum bending radius, respectively.

Small systematic anomalies are also visible in the large tailing radii domains, where the simulated values are marginally higher than experiment and decay more slowly. Their variant discrepancies are caused by idealization assumptions: the model assumes strict axial uniformity, but actual fibers have a perturbation of the refractive index and manufacturing-induced structural inhomogeneities that cause complex scattering^38^. The idealized geometry also has a tendency to overestimate mode field shift under bending and causes a slight inflation of predicted radiation losses^39^. However, the model can effectively model major tendencies in the bending loss, and the estimates are also conservative at small bending radii, which justifies it as a sound paradigm for exploring the effects of structural perturbations at large bending.

**Fig. 7:**
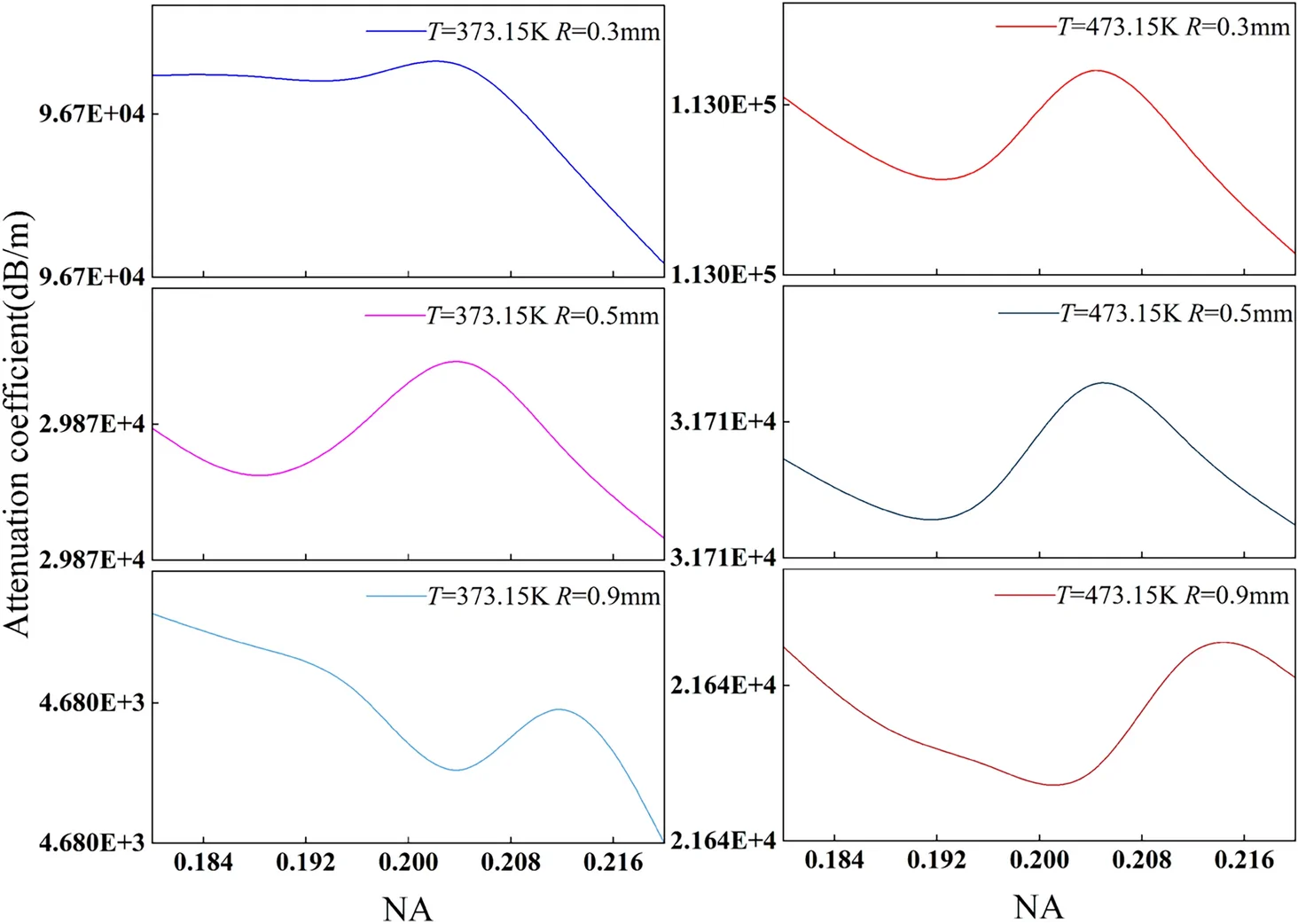
The temperature response of MMF microbend loss versus NA.

**Fig. 8:**
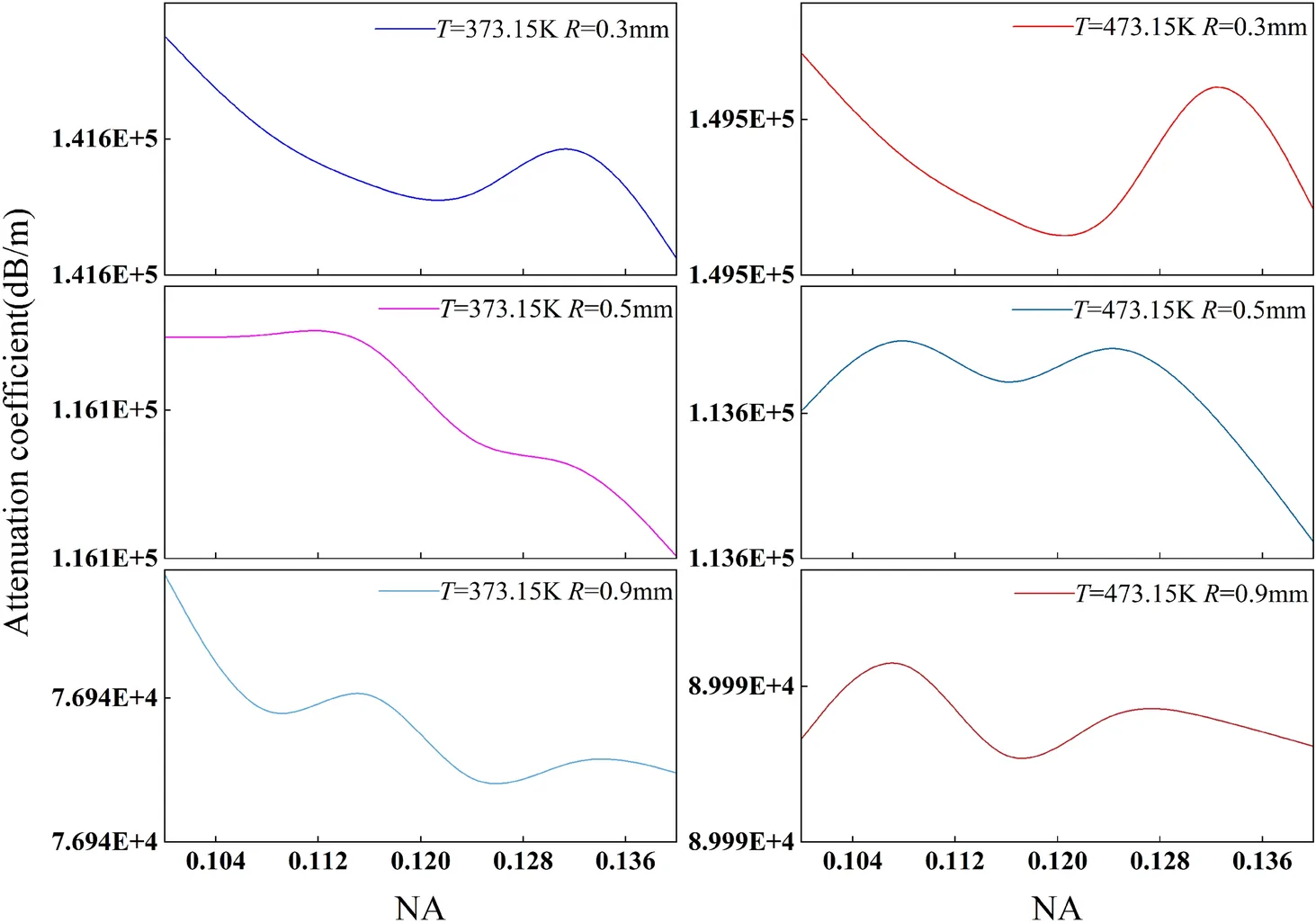
The temperature response of SMF microbend loss versus NA.

Figures 7 and 8 show the microbending loss responses of MMF and SMF at different temperatures and bending radii. With increasing *R* from 0.3 mm to 0.9 mm, the attenuation coefficients of the two types of fibers decrease sharply by about an order of magnitude, as expected with classical bending radiation theory. The additional study shows that the attenuation coefficient oscillates non-monotonically with the numerical aperture (NA), with loss peaks and troughs alternating.

This is in line with the modal coupling effects caused by bending as observed in^40^, which proves that the model has the ability to resolve coherent coupling between guided and cladding/radiation modes. A comparison of the results at *T* = 373.15 K and 473.15 K has shown a systematic temperature-dependent change in resonance peak positions due to the effect of variations of refractive index from the TOE, changing effective index contrast, and adjusting modal phase-matching conditions. These results indicate that the model has always been able to represent the synergistic effect of geometric bending and thermal variation of microbend loss.

To provide a clear boundary for the proposed model and prevent over-extrapolation, it is necessary to outline its assumptions and simplifications explicitly. Primarily, this remains a static (steady‑state) model. Via parameter sweeps, it assesses thermal response and loss traits at discrete equilibrium states—thus ignoring transient thermo‑mechanical coupling along with time‑dependent dynamic behaviors. Next, placing intrinsic optical coupling mechanics ahead of complex physical load transfers, the model adopts equivalent stress‑optic terms and equivalent curvature parameters.

Hence, we omit dynamic mechanical loads and secondary cladding mechanical effects. Lastly, simulations run on a 2D cross‑section using a uniform axial approximation. Such geometric simplification presumes adiabatic axial curvature variations along with single‑plane bending—a premise that may not wholly represent the three‑dimensional complex spatial deformations found in real downhole environments. These simplifications are deliberately chosen to secure computational convergence and physical interpretability for static micro‑bending cases.

**Fig. 9:**
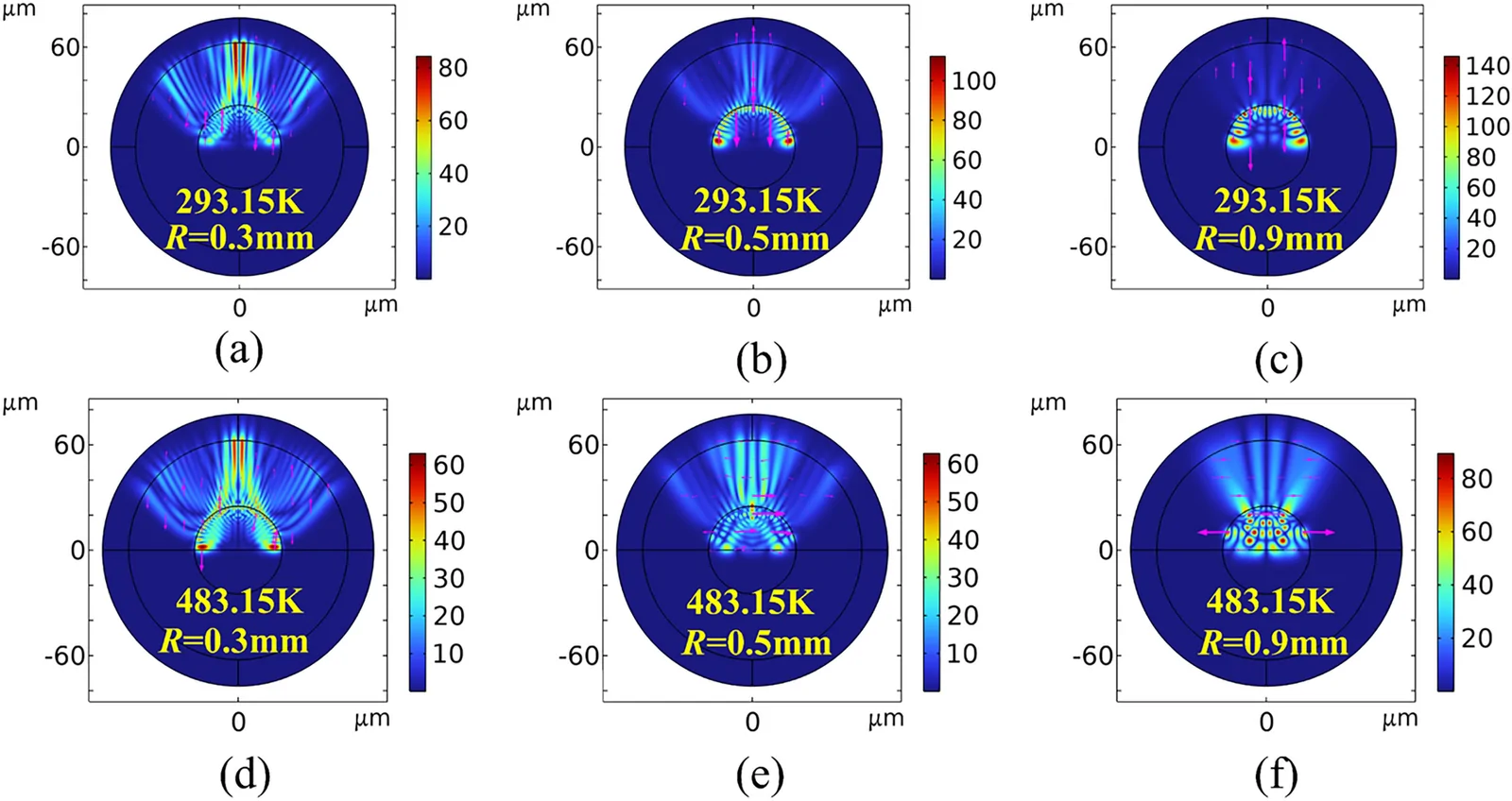
The comparison of electromagnetic field distributions for microbending loss in MMF at room temperature and 483.15 K (The blue regions represent the fiber matrix; brightness indicates electric field intensiy distribution; while magenta vector arrows indicate magnetic field vector direction.).

**Fig. 10:**
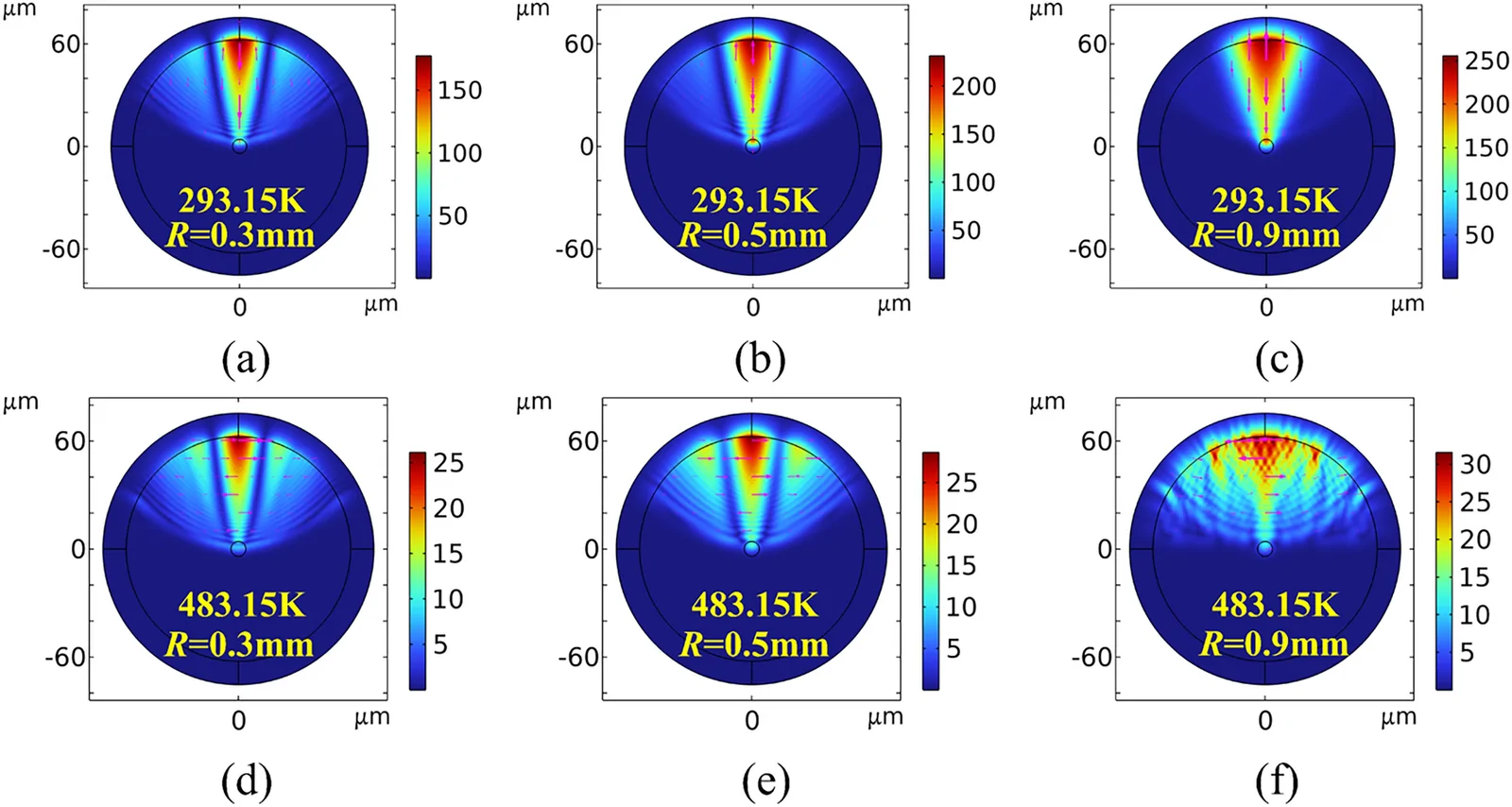
The comparison of electromagnetic field diagrams for bending loss of SMF at room temperature and 483.15K.

Figures 9 and 10 show the distributions of electromagnetic fields at the cross-section of the fiber at varying temperatures and bending radii. The smaller the bending radius, the more the mode field is redirected to the outer side of the bend, resulting in more intense radiative components in the cladding. This behavior indicates that bending-induced tilting of the equivalent refractive index distribution weakens the waveguide’s lateral confinement of the mode field. This trend is consistent with the physical interpretation of the equivalent refractive index model for bent waveguides^41^.

Temperature variations significantly affect the mode field distribution. Compared with room-temperature conditions, the mode field exhibits a more extended spatial distribution at elevated temperatures, indicating reduced waveguide confinement. This behavior is attributed to thermo-optically induced changes in the material refractive index, which modify the effective index contrast between the core and cladding and thereby affect lateral mode confinement^42^. The expanded mode field implies an increased coupling probability between guided and radiation modes, consistent with numerical results that show enhanced loss at higher temperatures.

Figure 10 shows the distribution of the electromagnetic field of SMF at various temperatures when bending. Just like in MMF, a higher bending radius delivers weaker radiation elements, but a higher temperature offers greater field growth and radiation. The equivalent refractive index gradient changes with temperature variations by changing the material refractive index distribution, which increases the loss of guided to radiation mode coupling and results in a net increase in loss^43^. The numerical findings indicate that the physical interpretation of equivalent results of the model is consistent at the microscopic loss scale, and at the level of microscopic field distributions, hence demonstrating that the model proposed is a coherent way of explaining the fiber microbending loss when the combined effect of thermal and bending is taken into account.

## Simulation analysis and discussion

### The temperature response of fiber microbend loss

**Fig. 11:**
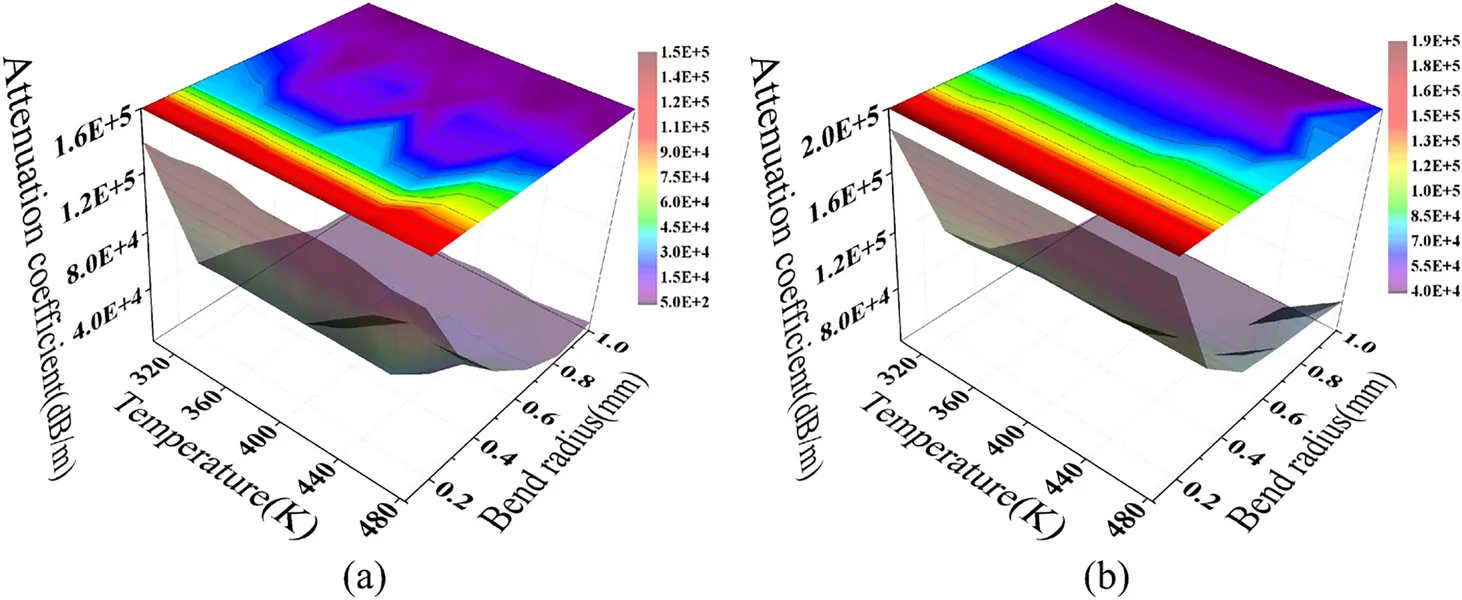
The temperature response of microbend loss for MMF and SMF, (**a**) the MMF model data, and (**b**) the SMF model data.

Figure 11 shows the distribution of attenuation coefficient in 3D over 303.15 ~ 480.15 K and bending radii of 0.1 ~ 1.0 mm. In the case of MMF, non-monotonic fluctuations along both axes of the attenuation surface in Fig. 11a is a strong manifestation of quasi-periodic fluctuations caused by changes in temperature of the propagation constant difference (Δ*β*) and the multimode beat length (*L*_b_), which means that the loss is largely controlled by modal phase-matching conditions. Microbending is greatly effective in increasing the coupling between guided and radiation modes on a small radius (*R* ≤ 0.4 mm) circuit, making attenuation very sensitive to changes in geometry. Over larger radii (*R* ≥ 0.6 mm), the strength of coupling and the preeminence of modal phase-matching evolution are both reduced, with the TOE determining the positions and sizes of loss variations.

Conversely, the SMF attenuation response in Fig. 11b has a monotonic, almost-linear dependence on both temperature and bending radius, indicating loss that is dominated by direct radiation leakage of the fundamental mode. Although attenuation is not very sensitive to microbending at low temperatures and high radii, it increases very quickly as one gets into the high-temperature regime (*T* ≥ 440 K). This drastic value enhancement is a result of the synergistic interactions of the thermo-optically induced changes in refractive indices and the mode field expansion, two systems that weaken transverse confinement. Physically, it is represented by the decrease of normalized frequency *V*, which reduces the waveguide guiding capacity and promotes leakage of fundamental modes in the air.

These 3D distributions show that there is a basic difference between temperature-microbend coupling mechanisms in MMF and SMF. In the case of SMF, the loss response to microbending is greatly escalated at high temperatures (*T* ≥ 440 K) and this is a key feature that shows that it can be sensitive to the conditions of mechanical isolation at high temperature; as a monotonic attenuation response, it is also suitable to high-speed telemetry with sufficient structural protection and further demonstrates the high reliability of signal distinguishability to detect anomalies based on a threshold^44^. On the other hand, MMF has oscillatory loss responses that reflect high tolerance to random structural perturbations. This robustness makes MMF more suitable for short-distance transmission under harsh downhole conditions^45^. However, the loss fluctuations induced by modal interference may compromise measurement stability in high-precision distributed sensing applications.

### The spectral analysis of temperature response in fiber microbend loss

**Fig. 12:**
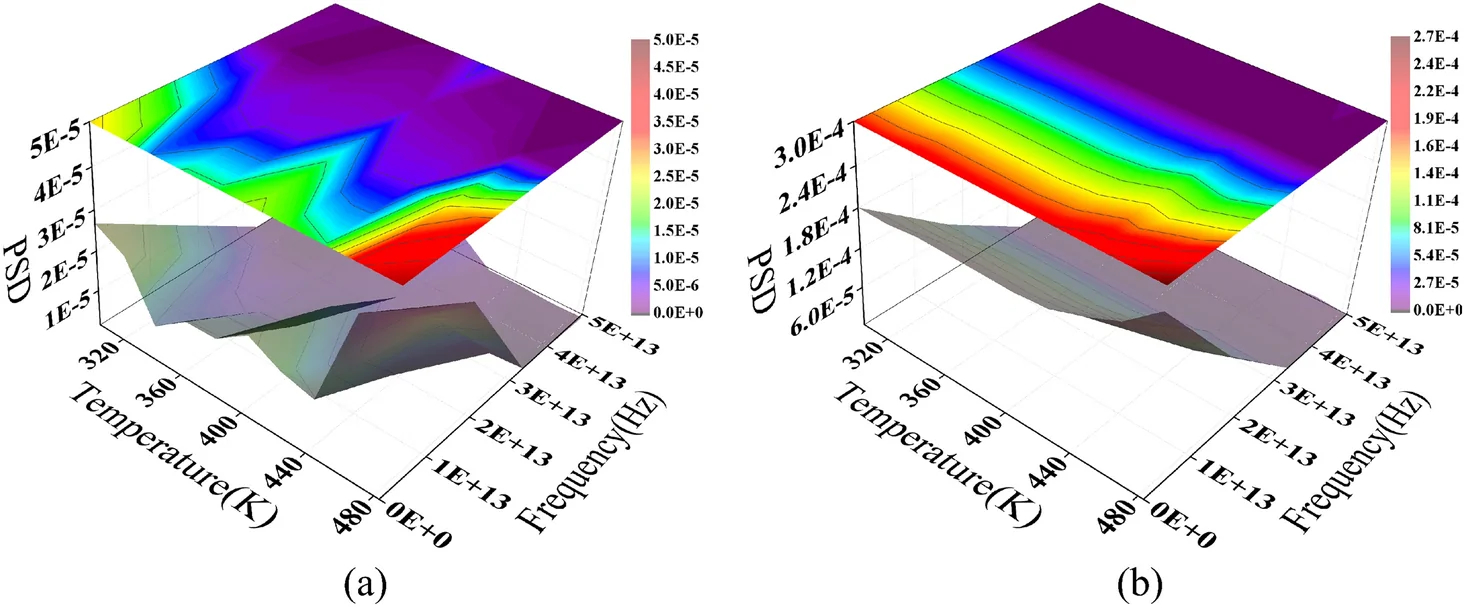
The temperature-dependent PSD of fiber microbend loss, (**a**) MMF, and (**b**) SMF.

The periodic nature of microbend loss was analyzed using a 3D PSD approach based on the temperature and bending radius parameter space (Fig. 12). By processing the discrete attenuation coefficient sequences, the PSD characterizes the characteristic scales and intensities of the loss fluctuations. Notably, the frequency axis in Fig. 12 represents the optical spectral frequency, characterized by a 10^13^ Hz scale. Given that the 1.55 μm center carrier corresponds to an optical frequency of approximately 193 THz, the 10 THz component represents the spectral modulation intensity induced by the TOE on the optical field envelope, rather than the electrical modulation bandwidth of the communication system.

The spectral characteristics of the MMF and of SMF are different in essence. The MMF PSD (Fig. 12a) is multi-peaked with stochastic variation, which is due to the interaction between multimode interference and temperature-induced change in propagation constants which results in multiscale phase modulation between guided modes and offers an imperative clarification to modal noise in real-world fiber systems.

On the other hand, the SMF PSD (Fig. 12b) displays a smooth profile with monotonically decreasing amplitude that rises progressively with temperature, which confirms the fact that the loss is caused by fundamental-mode leakage enabled by mode-field expansion. Above 440 K, the TOE reduces the normalized frequency *V*, expanding the mode field and enhancing radiation leakage, which greatly amplifies PSD. It is interesting to note that the highest SMF PSD amplitude is almost an order of magnitude greater than that of MMF (10^−4^ compared to 10^−5^), which is a manifestation of the highly sensitive and signal-unstable behavior of SMFs in high-temperature, microbending-prone conditions.

These divergent spectral characteristics have direct engineering implications. The main limiting factors that affect multimode systems are intensity noise due to modal interference, whereas the SMF systems are more susceptible to variations in link attenuation. Quasi-periodic PSD modulations of MMFs can lead to a reduction of signal stability with a change of temperature in favor of non-coherent modulation or speckle suppression techniques. The large amplitude SMF PSD response at high temperature needs complex receiver dynamic range and gain control. Enhancement of the mechanical isolation of the fiber-optic cable is also an advisable method of controlling such a basic mitigation technique of maintaining optical link budgets in high-temperature deep wells^46^.

**Fig. 13:**
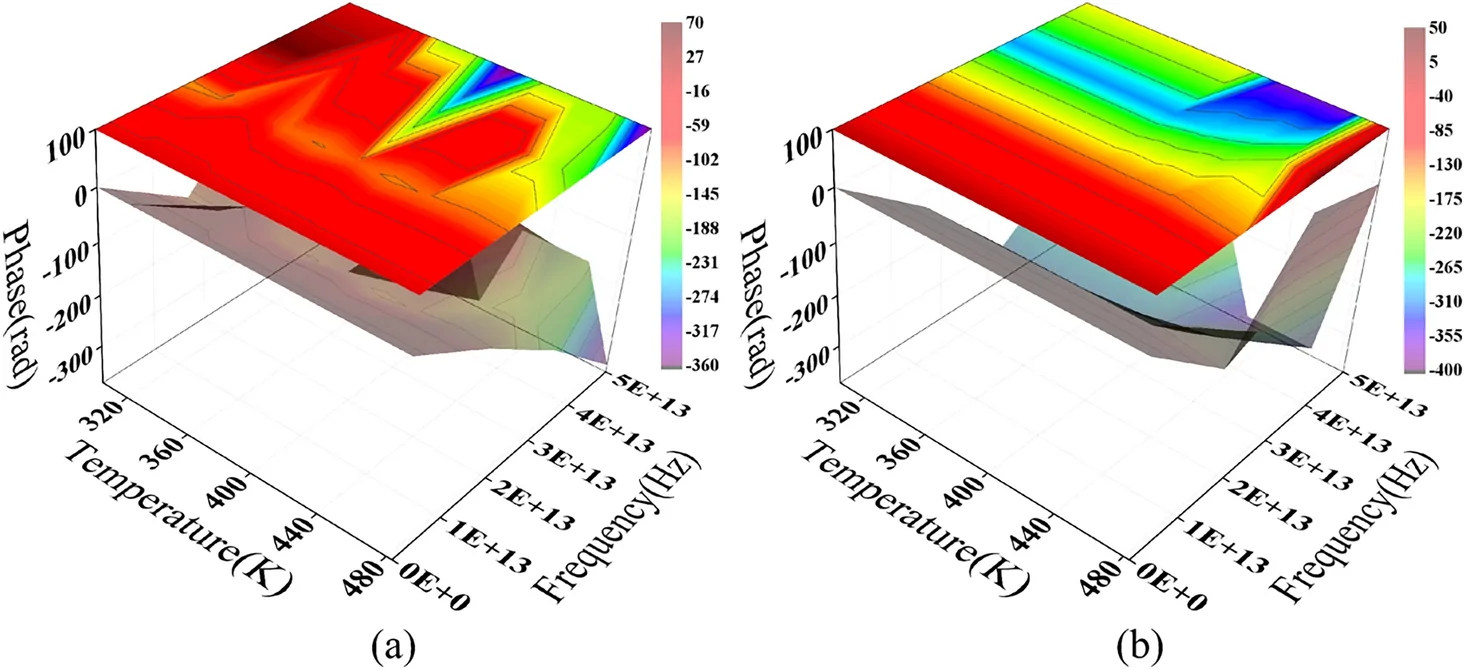
The temperature-dependent phase spectrum of fiber microbend loss, (**a**) MMF; and (**b**) SMF.

The phase response of optical field mapping is plotted against frequency-temperature parameter space (Fig. 13), which shows significantly different wavefront development of the two fiber types. The discontinuous jumps and local topological transitions of the MMF phase distribution (Fig. 13a) shows that strong complex amplitude modulation is due to the multimode interference evolution under different temperature and microbending conditions. In cases where the propagation constant difference between guided modes changes substantially, the composite field vector varies substantially in the immediate vicinity of the point of origin in the complex plane, appearing as phase singularities that have a typical 2p phase jump^47^. It means that with mixed thermal and structural perturbation, inter-modal coupling in MMFs is highly sensitive to parameters, and this relationship worsens optical field coherence.

Conversely, the SMF phase spectrum (Fig. 13b) has a continuous and smooth gradient distribution due to optical path length modification, caused by TOE. The propagation constant variation is deterministic such that the accumulation of the phase of the fundamental mode is monotonically continuous. Strongly, although the attenuation of the amplitude in the corresponding areas of Fig. 12 is very high, no phase singularity is observed in Fig. 13b, which proves that the fundamental-mode wavefront does not experience the topological change. This allows SMF to maintain large phase discernibility in highly attenuating conditions and gives it a solid physical basis for coherent detection in extreme conditions.

High sensitivity to environmental disturbances that can destabilize coherent demodulation is manifested by the phase discontinuities at high levels in MMFs, which are sensitive to environmental disturbances^48^. Conversely, the phase continuity and topological robustness exhibited by SMFs facilitate reliable coherent sensing, even in strongly attenuating regimes. In challenging downhole environments (high temperature and high pressure), the deterministic phase evolution of SMFs significantly improves the discrimination of strain, acoustic, and thermal signals, provided that sufficient power budgets are available.

### The effect of operating wavelength on the temperature response of fiber microbend loss

**Fig. 14:**
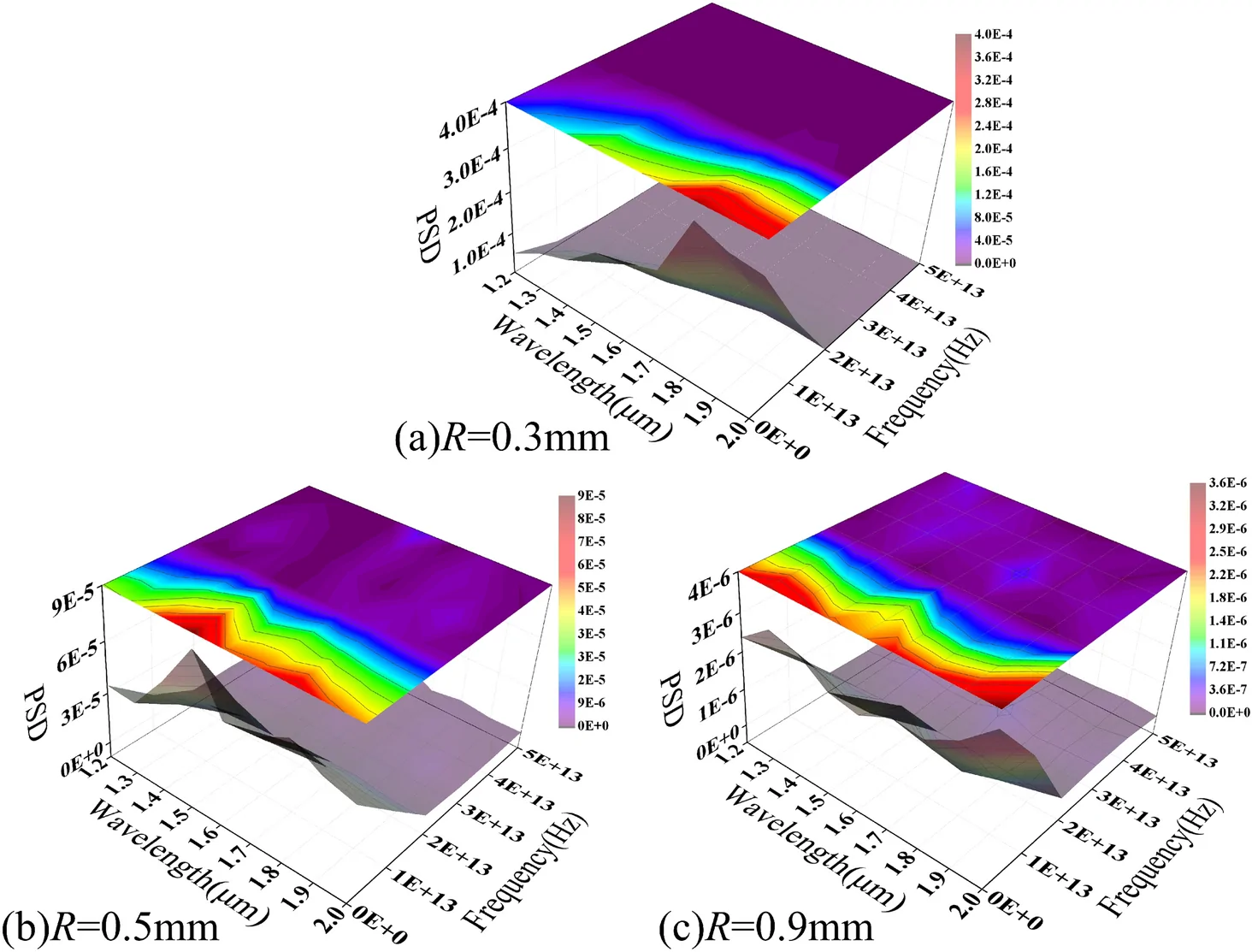
The PSD of the temperature response of microbend loss as a function of wavelength in the MMF model.

Figure 14 illustrates the distribution characteristics of the PSD for MMF microbending loss under thermo-microbend coupling conditions across the frequency-wavelength parameter domain, with bending radii *R* set to 0.3, 0.5, and 0.9 mm. The PSD is computed via a frequency-domain transformation of the temperature-induced loss sequences to identify the characteristic scales and energy distribution of loss fluctuations under parameter perturbations. The results indicate that the overall PSD amplitude decreases with increasing wavelength and bending radius. Furthermore, the spectral structure exhibits broadband and non-periodic distribution characteristics across all investigated bending radii.

It is worth noting that although the PSD has a clear change in amplitude and local energy distribution between different bending radii, it is clear that the spectral structure of the PSD always possesses aperiodic features across its broadband spectrum range. This invariance implies that it is the bending radius that mostly changes the coupling strength and does not change the underlying stochasticity of multimode random coupling. Therefore, spectral morphology can be used as a marker of the type of coupling mechanism, and spectral amplitude and distribution details can be used to describe the intensity of perturbation and the development of the phase-matching windows.

The analysis of PSD shows that the phenomenon of temperature-dependent microbending loss is stochastically coupled in a frequency domain point of view, and shows that fluctuations at the wavelength scale are largely determined by the interaction between the density of modes, phase-matching based on the distribution of curvature perturbation. It is a unified physical description of the MMF loss behaviour under coupled curvature, wavelength, and thermal perturbation, and this has formed a strong parameterization foundation on which to base the performance evaluation of the system at a later stage.

**Fig. 15:**
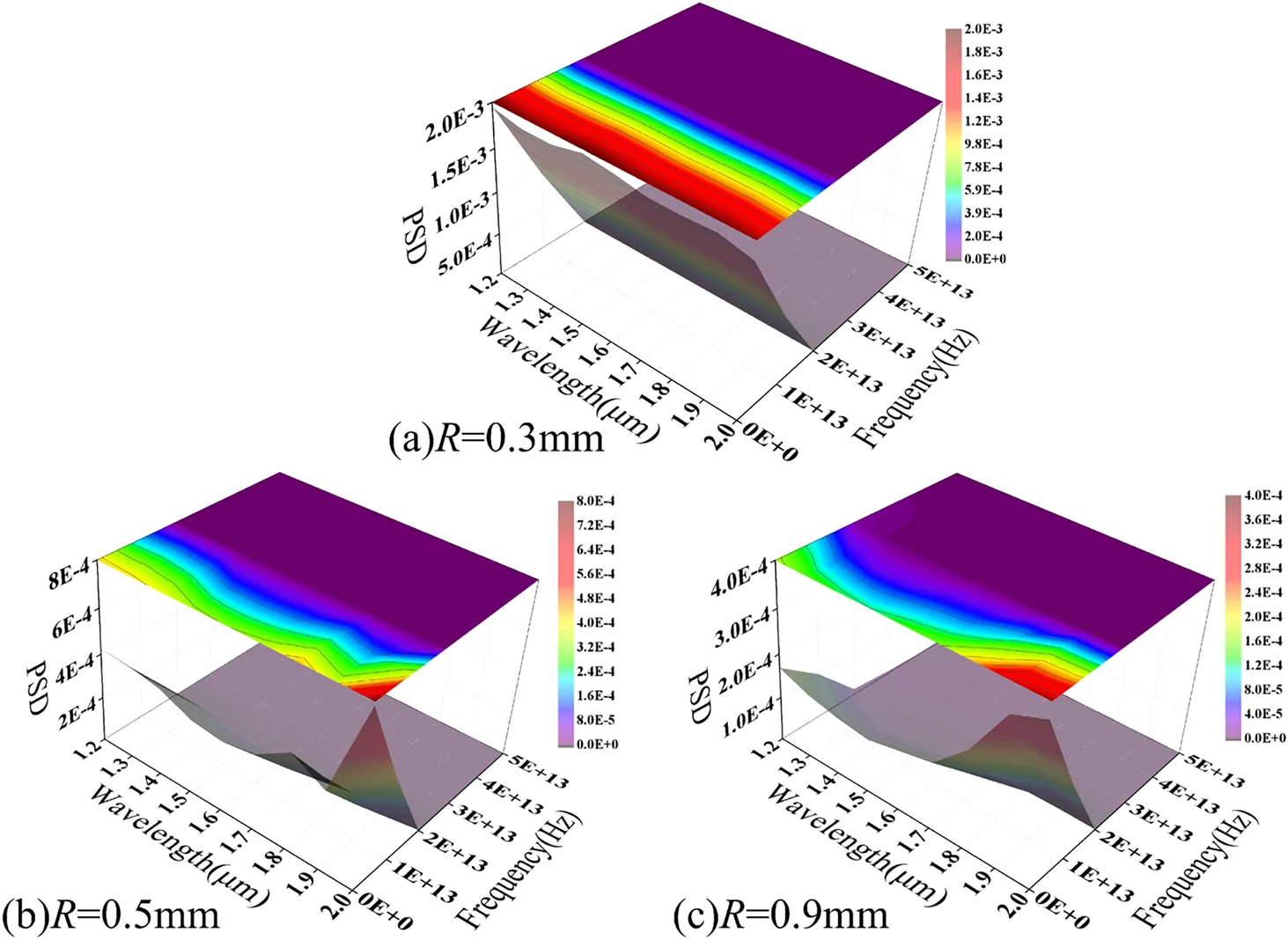
The PSD of the temperature response of bending loss as a function of wavelength in the SMF model.

Figure 15 shows that with thermo-microbend coupling, the SMF loss response is highly enhanced with increased wavelength and has unique sensitivity at long wavelengths. In comparison to the statistical inter-modal interference mechanism used in MMF, the SMF PSD increases at a high rate at longer wavelengths. At operating wavelengths above about 1.6 μm, the normalized frequency *V* is reduced substantially, and fundamental mode transverse confinement is lost; this will cause the mode to begin expanding into the cladding and become more sensitive to microbending perturbation. This is mostly amplified at smaller bending radii (*R* = 0.3 mm) where the amplitude of PSD is much larger than that of MMF, suggesting that one has entered a radiation-leakage loss-enhancement regime.

It is also seen in comparing Figs. 14 and 15 that the natural difference in spectral response mechanisms is that loss in SMF is largely controlled by the strength of transverse confinement, and loss in MMF is controlled by the stochastic character of modal interference. Engineering-wise, shorter wavelengths provide more stable transmission in the situation of severe thermo-mechanical conditions, whereas longer wavelength bands (their high sensitivity to structural perturbations) can have potential applications in deformation and anomalies detection. These are the conclusions that provide a physical basis for the wavelength choice and structural optimization of subsurface fiber optics.

**Fig. 16:**
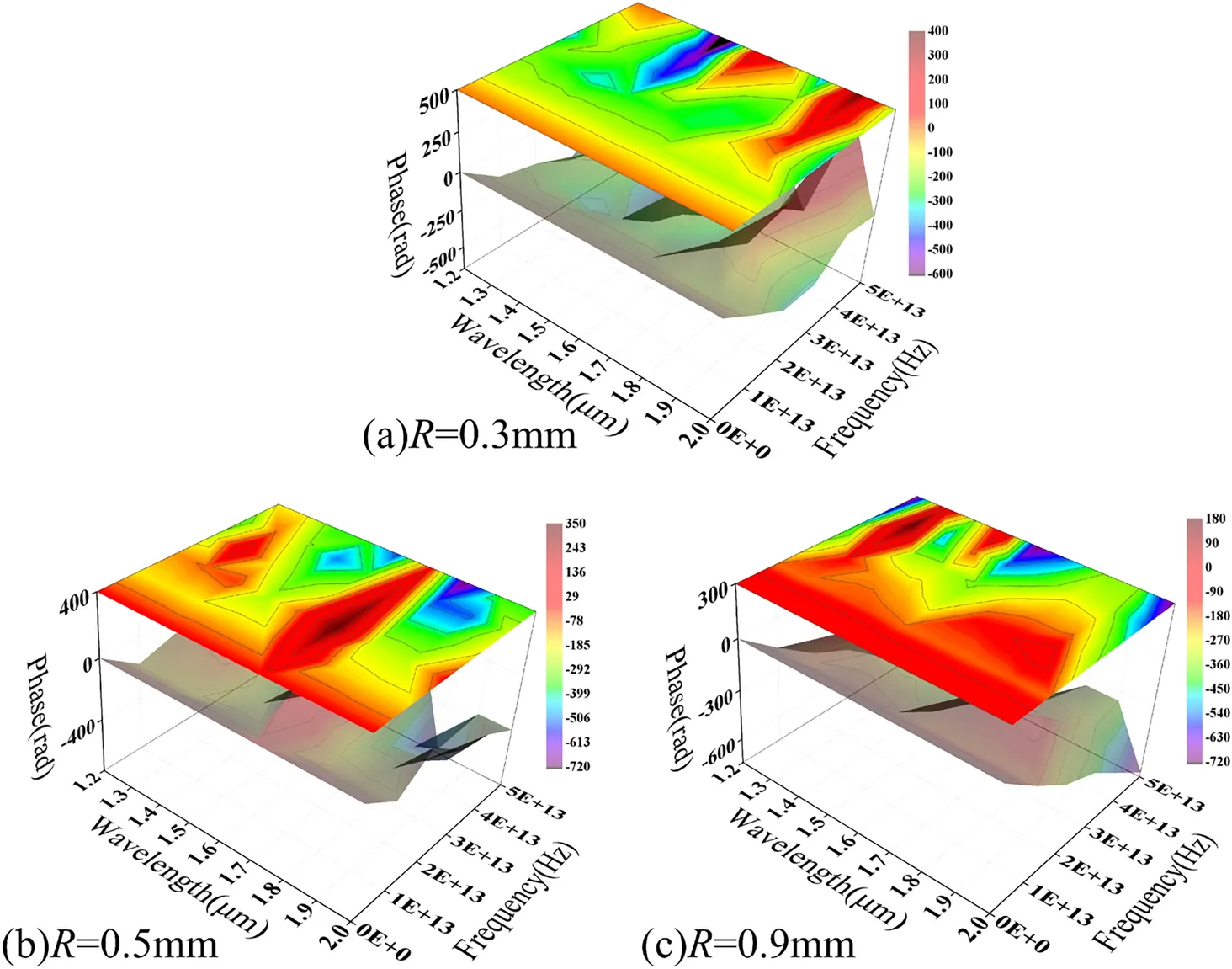
The phase spectrum of the temperature response of microbend loss as a function of wavelength in the MMF model.

The phase response spectra of MMF under thermo-microbend coupling are shown in Fig. 16. The production stage field has a high level of spatial decorrelation and local discontinuities, which are based on spectral phase-shifting through intermodal dispersion. Owing to the variation in dispersion group reactions among guided modes (∂*β*_m_/∂*λ* ≠ ∂*β*_n_/∂*λ*), minor changes in wavelength cause nonlinear growth of relative inter-modal phase, which restructures the multimode interference (MMI) structure on a rapid basis^49^. With tight-bending (*R* = 0.3 mm), higher-order mode (HOM) participation is boosted by the fact that the lateral perturbations and their resulting complex amplitude tend to go towards zero in localized regions, and that topological singularities are the result of creating phase jumps of 2π.

According to the complex coherence factor *μ* defined by Eq. (13), the inclusion of HOMs under thermo-microbending coupling, combined with intermodal dispersion-induced phase shifts, renders the phase at discrete spatial points stochastic. When statistically averaging the random speckle patterns, the product *E*(*x*)E*(*x* + Δ*x*) approaches zero due to phase randomization (where *E*(*x*) is the complex amplitude and E*is its conjugate). Consequently, |*μ*(Δ*x*)| decays sharply toward zero as Δ*x* increases, with the decay rate corresponding to a shortening of the spatial correlation length. This demonstrates the statistical coherence degradation of multimode fields, where the spatial correlation length of the output wavefront is significantly reduced, and phase stability markedly deteriorates.

13 $$\mu (x) = \frac{{\left\langle {E(x) \cdot E^{*} (x + \Delta x)} \right\rangle }}{{\sqrt {I(x) \cdot I(x + \Delta x)} }}$$

These findings suggest that when the thermal-microbending effects are uncoupled, the transmission of MMFs is more and more stochastically coupled, so there is less phase to recover. This phase-decorrelation phase considerably raises the stability needs of coherent demodulation, as well as produces essential limitations to broadband phase-sensitive sensing. Therefore, MMFs can be used more effectively in intensity-based or non-coherent modulation schemes in complicated downhole conditions.

**Fig. 17:**
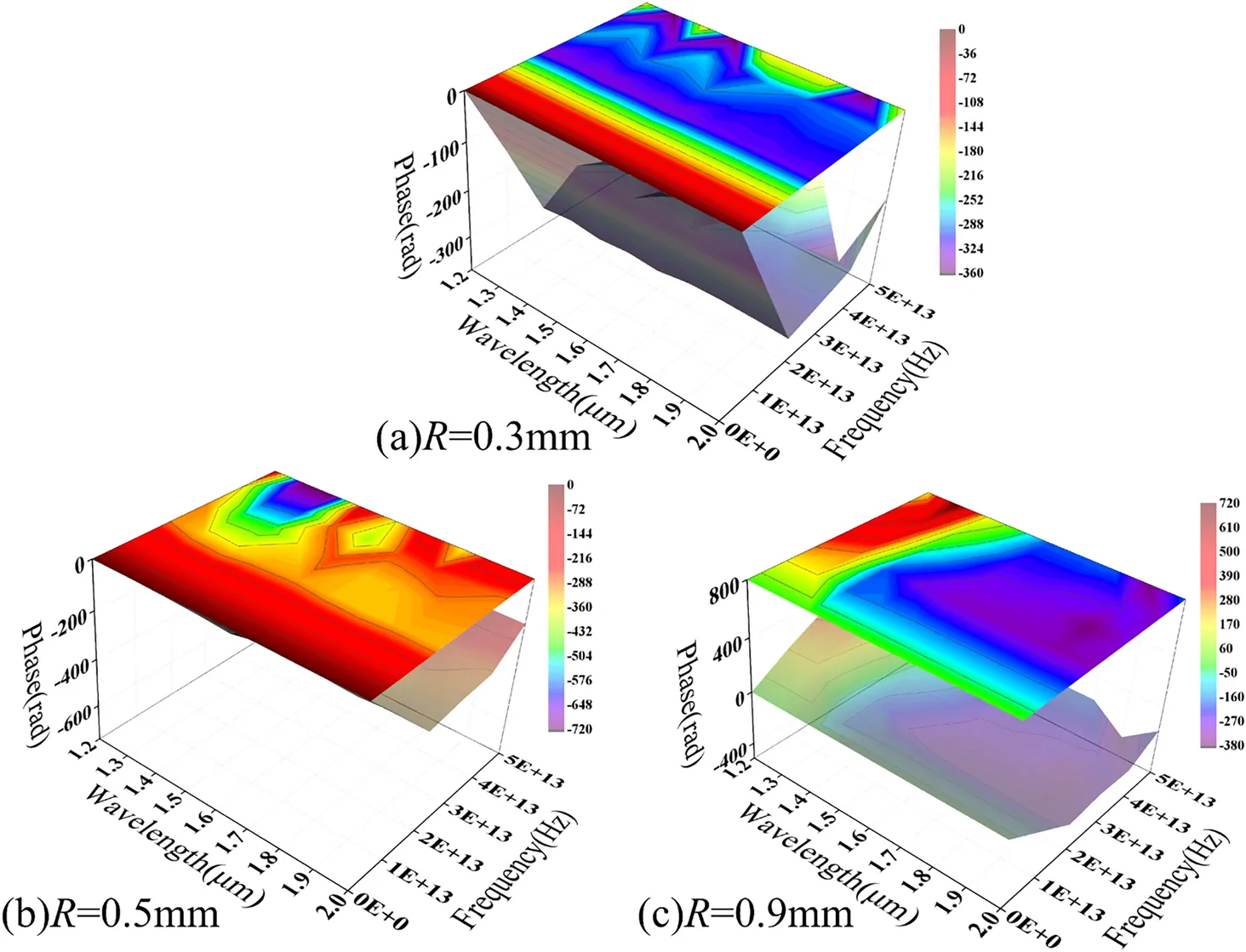
The phase spectrum of temperature-dependent microbend loss in the SMF model as a function of wavelength.

Figure 17 represents the phase response development of SMF when there are coupled thermal and microbending conditions. Compared to MMF, the SMF has strong phase continuity throughout the whole parameter space and has no phase singularities or topological discontinuities, with fluctuations in the frequency axis remaining below 20%. This stability is because of the analytical determinism of single-mode propagation, wherein the fundamental mode phase is given as:

14 $$\varphi \, = \, \frac{2\pi }{\lambda }n_{eff} \left( {T,R,\lambda } \right)L(R)$$

In this context, *n*_eff_ is a continuous function of temperature *T*, bending radius *R*, and *λ*, while *L*(*R*) denotes the effective optical path length. Owing to the absence of phase cancellation typically induced by multipath interference, the residual propagating mode maintains a well-defined wavefront topology even in high-loss regimes^50^. This structural stability underscores the deterministic nature of the single-mode phase evolution under extreme perturbations.

At high bending radius (with increasing bending radius), the prevailing physical processes undergo a gradual change between 0.3 mm and 0.9 mm. At tight-bending (*R* ≤ 0.5 mm), the phase modulation depends mainly upon the geometric path effects that arise due to bending and the stress birefringence. The weakening of curvature (*R* ≈ 0.9 mm) leads to reduced contribution of geometry and gradually progresses towards an intrinsic material-dominated phase response, giving rise to a dispersion-like evolution which is defined by thermo-optic effects. Such a transition allows one to decouple structural perturbations and intrinsic thermal signatures in the weak microbending range.

Phase and loss spectral analysis just shows that although the amplitude may experience exponential decay in the long wavelength regime, phase continuity can still be maintained provided the propagating mode meets the minimum detectable SNR. This separation of amplitude decay and phase retention establishes the SMFs as predicted to maintain recoverable deterministic phase information in the condition of extreme thermo-microbend coupling, which gives a mechanistic basis for coherent distributed sensing and interferometry. However, as of practical implementation, optical link power budgets and mechanical disturbance suppression are limited.

## Conclusion

To mitigate signal attenuation caused by fiber micro-bend loss in high-temperature deep-well environments (303.15 ~ 483.15 K), this study proposes a thermo-mechanical-optical coupling model based on equivalent refractive index modulation. The model explicitly integrates the TOE into the micro-bend perturbation framework, facilitating a unified description of fiber micro-bend loss under thermal extremes. Within the examined parameter range, the derived thermo-optic coefficient (~ 10^−5^ K^−1^) is consistent with literature values. Simulation results demonstrate that temperature does not act as a linearly superimposed, independent loss term. Instead, by modulating the core-cladding refractive index profile, temperature reconfigures waveguide confinement and alters the mechanisms of mode coupling and radiation leakage, thereby driving the nonlinear evolution of total attenuation.

MMF and SMF exhibit fundamental differences in their quantitative responses. In MMF, loss is governed by inter-modal phase matching, exhibiting oscillatory and non-monotonic behavior as a function of temperature and bending radius. Conversely, SMF demonstrates threshold-type behavior, with a significant loss transition occurring at temperatures *T* ≥ 440 K. The underlying mechanism is the enhanced radiative leakage driven by thermally induced mode-field expansion. Spectrally, the SMF PSD amplitude reaches 10^−4^, an order of magnitude higher than that of MMF (10^−5^). This indicates that SMF possesses higher environmental sensitivity under coupled high-temperature and micro-bend conditions, whereas MMF demonstrates greater tolerance to structural perturbations.

Phase spectra further highlight the distinct coherence properties of the two fiber types. Under stochastic mode coupling, MMF exhibits phase decorrelation and topological discontinuities, leading to diminished coherence stability. Conversely, SMF maintains continuous phase evolution even under high-loss conditions, with fluctuations remaining below 20%, demonstrating superior coherence retention. This decoupling behavior, characterized by "amplitude degradation versus phase preservation," provides the physical basis for coherent detection in extreme environments.

From an engineering perspective, this study quantifies the relationship between temperature (303.15 ~ 483.15 K), bending radius (0.1 ~ 1.0 mm), and operating wavelength. Long wavelengths (> 1.6 μm) significantly enhance SMF sensitivity to micro-bends. Conversely, stochastic coupling in MMFs limits the amount of phase information that can be stably recovered, thereby constraining the feasibility of broadband phase-sensitive sensing and multiplexing. Consequently, MMFs are better suited for short-distance, intensity-modulated data transmission or single-signal telemetry in downhole environments. In contrast, SMFs provide a viable solution for exploring deeper and more complex oil and gas reservoirs.

High-pressure effects on elastic constants and photoelastic coefficients are currently incorporated only indirectly via equivalent stress terms. An explicit pressure-dependent relationship for parameter evolution has yet to be established. In ultra-deep wells, pressure may influence micro-bend disturbance intensity by altering material mechanical properties and coating creep behavior. This mechanism represents a critical foundation for future model extensions.

This study presents a static theoretical analysis grounded in numerical simulations. Assuming a homogeneous quartz medium and equivalent curvature, the model is primarily applicable to axial gradual bending scenarios. It does not explicitly account for viscoelastic effects or load transfer mechanisms within the coating and cable structure under HTHP conditions. Consequently, the results provide a lower-bound estimation of the fiber’s intrinsic loss. Future research will incorporate pressure-dependent parameter evolution, multi-layer coupling, and experimental calibration to enhance predictive accuracy and engineering applicability in complex downhole environments. Furthermore, subsequent studies will place greater emphasis on rigorous experimental validation.

## Data Availability

The data presented in this study are openly available in OF-microbending-T at: https://github.com/08blackhorse/OF-microbending-T
